# Nitrogen Metabolism in Adaptation of Photosynthesis to Water Stress in Rice Grown under Different Nitrogen Levels

**DOI:** 10.3389/fpls.2017.01079

**Published:** 2017-06-23

**Authors:** Chu Zhong, Xiaochuang Cao, Jijie Hu, Lianfeng Zhu, Junhua Zhang, Jianliang Huang, Qianyu Jin

**Affiliations:** ^1^National Key Laboratory of Rice Biology, China National Rice Research InstituteHangzhou, China; ^2^Crop Physiology and Production Center, Huazhong Agricultural UniversityWuhan, China

**Keywords:** water deficit, nitrogen level, photosynthesis, nitrogen assimilation, rice (*Oryza sativa* L.)

## Abstract

To investigate the role of nitrogen (N) metabolism in the adaptation of photosynthesis to water stress in rice, a hydroponic experiment supplying with low N (0.72 mM), moderate N (2.86 mM), and high N (7.15 mM) followed by 150 g⋅L^-1^ PEG-6000 induced water stress was conducted in a rainout shelter. Water stress induced stomatal limitation to photosynthesis at low N, but no significant effect was observed at moderate and high N. Non-photochemical quenching was higher at moderate and high N. In contrast, relative excessive energy at PSII level (*EXC*) was declined with increasing N level. Malondialdehyde and hydrogen peroxide (H_2_O_2_) contents were in parallel with *EXC*. Water stress decreased catalase and ascorbate peroxidase activities at low N, resulting in increased H_2_O_2_ content and severer membrane lipid peroxidation; whereas the activities of antioxidative enzymes were increased at high N. In accordance with photosynthetic rate and antioxidative enzymes, water stress decreased the activities of key enzymes involving in N metabolism such as glutamate synthase and glutamate dehydrogenase, and photorespiratory key enzyme glycolate oxidase at low N. Concurrently, water stress increased nitrate content significantly at low N, but decreased nitrate content at moderate and high N. Contrary to nitrate, water stress increased proline content at moderate and high N. Our results suggest that N metabolism appears to be associated with the tolerance of photosynthesis to water stress in rice via affecting CO_2_ diffusion, antioxidant capacity, and osmotic adjustment.

## Introduction

Nitrogen (N) fertilizer plays a vital role in yield increasing of major food crops worldwide during the second half of the 20th century (Chardon et al., 2012; Lassaletta et al., 2014). As diminishing returns of increasing investment of N fertilizer, excessive application of N fertilizer causes stagnation of crop yield and lowers nitrogen use efficiency (NUE) of crops (Lawlor, 2002). Photosynthesis is recognized as one of the most efficient ways to increase NUE and crop yield (Kumar et al., 2006). Zhu et al. (2010) have proposed that further increases in crop yield potential will rely in large part on improved photosynthesis. However, improvement of photosynthesis in field conditions confronts some challenges, and the most influential one is seasonal and regional water stress. The exposure of plants to water-limiting conditions results in the decreases in gas exchange and chlorophyll fluorescence triggered by increased resistance to CO_2_ diffusion and metabolic constraints (Chaves and Oliveira, 2004; Pinheiro and Chaves, 2011). To better understand the underlying physiological mechanisms that plant photosynthesis in response to reduced water availability and how these can be manipulated are essential to improve plant photosynthetic capacity.

The majority of assimilated N in plant is invested in photosynthetic machinery (Nunes-Nesi et al., 2010). Therefore, N is fairly strong positive correlated with photosynthetic rate (Makino et al., 2000, 2003). In addition to directly taking part in photosynthesis, N acts as an important regulator in manipulation of CO_2_ diffusion as indicated by the strong positive correlation between N and stomatal conductance (*g*_s_) or mesophyll conductance (*g*_m_) (Tosens et al., 2012; Xiong et al., 2015). In fact, nitrogenous compounds in and outside plant play an essential role as signals in regulating the responses of plants to environmental changes (Tabuchi et al., 2007; Gojon et al., 2009). It has been well documented that nitrate uptake, allocation, and assimilation in plants were closely associated with the resistance of plants to adverse conditions including water stress (Chen et al., 2012; Atkinson et al., 2014; Fang et al., 2016).

The regulatory function of N in water stress tolerance of plant depends upon the intensity of stress and N level. Proper N level supports regular plant growth and helps plants to defense stress (Chang et al., 2016). It has been proposed that crops supplied with relative higher N had better growth performance than that supplied with low N under drought stress (Haefele et al., 2008; Tran and Yamauchi, 2014; Wang et al., 2016). Higher N mitigates the adverse effects of water stress on photosynthesis as well. Otoo et al. (1989) revealed that relative to low N, higher N increased the sensitivity of *g*_s_ to water stress and maintained better photosynthetic machinery in rice. Increased resistance of photosynthesis to water stress at higher N also resulted from improved enzymatic and/or non-enzymatic antioxidant systems (Chang et al., 2016). Besides, more N is allocated to soluble organic nitrogenous compounds, such as proline and other amino acids under higher N levels, which act as osmotica to balance water status in plant cell under osmotic stress (Funk et al., 2013; Singh et al., 2016).

Nitrogen metabolism regulation is crucial for stress tolerance and involves almost all physiological processes in plants (Lawlor, 2002). Water stress induced reduction in photosynthesis was associated with the decline in N metabolism (Garg et al., 2001; Xu and Zhou, 2005, 2006). Glutamine synthase (GS) and glutamate synthase (GOGAT) are the key enzymes for incorporation of inorganic N into amides and amino acids in cell, bridging carbon and nitrogen metabolism. Singh et al. (2015) demonstrated that soil drying reduced the activities of these enzymes as well as N uptake and contents of nitrogenous compounds in leaves of sunflower seedlings. Ammonium is a central intermediate product of nitrogen metabolism in plants. It derives from external source by root uptake and reduction of nitrate, or from internal source by degradation of amino acids and photorespiration (Thomsen et al., 2014). As high concentration of ammonium is toxic to plant cell, the high capacity to assimilate ammonium could be an important factor in alleviating the consequence of stress (Hoai et al., 2003). GS involves in the process of ammonium assimilation and reassimilation (Miflin and Habash, 2002). It has been recognized as a metabolic indicator of drought stress tolerance in wheat (Nagy et al., 2013). The cytosolic (GS1) and chloroplastic (GS2) isoforms of GS catalyze the assimilation of ammonium derived from different sources (Miflin and Habash, 2002; Thomsen et al., 2014). It has been reported that the activities of GS1 and GS2 isozymes varied substantially in different water-sensitive cultivars of rice when exposed to water stress (Singh and Ghosh, 2013). This suggests that the different tolerance of rice cultivar to water stress may partly attribute to ammonium assimilation and/or reassimilation (Hoai et al., 2003). However, further research is needed on how N metabolism contributes to water stress tolerance in rice.

Rice (*Oryza sativa* L.) is a water intensive crop. For example, in Asia, where rice cultivation area accounts for over 90% of the world, rice cultivation consumes up to 80% of agricultural irrigation water (Bouman and Tuong, 2001). Developing water-saving irrigation in rice production is of great significance to sustainable development of agriculture. Water-saving irrigation, e.g., alternate wetting and drying (AWD), alters the relationship between water and nitrogen status in the field, which may affect fundamental biochemical processes and eventually grain yield in rice plants (Dodd et al., 2015; Wang et al., 2016). The aim of this study was to examine the role of N metabolism in the adaptation of photosynthesis to water stress in rice.

## Materials and Methods

### Plant Material and Growth Condition

Seeds of hybrid *indica* rice cultivar ‘Zhongzheyou 1’ were germinated and grown hydroponically in a nutrient medium described as follows. At third leaf stage, the seedlings were started to be supplied with 1/2 strength nutrient solution. At sixth leaf stage, the seedlings were transplanted to 5-L pots with sponge wrapped around the interface of the root and the shoot and cultivated with nutrient medium. The full-strength nutrient medium contained 1.43 mM NH_4_NO_3_ (2.86 mM N), 0.32 mM NaH_2_PO_4_, 0.5 mM K_2_SO_4_, 1.0 mM CaCl_2_, 1.7 mM MgSO_4_, 9.1 mM MnCl_2_, 0.52 μM (NH_4_)_6_Mo_7_O_24_, 18.0 μM H_3_BO_3_, 0.15 μM ZnSO_4_, 0.16 μM CuSO_4_, 36.0 μM FeCl_3_, and 70 μM citric acid. Additional 50 mL concentrated sulfuric acid and 10 g EDTA-Na_2_ were added in per liter of micronutrient mixture solution.

At the first 4 days after transplanting, seedlings were supplied with 1/2-strength nutrient solution (1.43 mM N), and then followed by supplied with full-strength nutrient solution (2.86 mM N) during the next 10 days. After N starvation for 1 day, seedlings were separated into three groups, and provided with 0.72 mM N (low N), 2.86 mM N (moderate N), and 7.15 mM N (high N), respectively, for additional 10 days until the occurrence of significant phenotypic difference in plant size and leaf color. In the following 5 days of N treatment, half of the plants in each treatment were treated with 150 g⋅L^-1^ PEG (6000) as water deficit (WD), and with no PEG (6000) as control (well-watered, WW). The solution was refreshed twice a week to maintain a pH of 5.50 ± 0.05.

The experiment had six treatments (three N levels and two water conditions), and each treatment had four independent pots, each of which consisted of four independent plants. The experiment was arranged in a completely randomized design in a factorial (3 × 2). The position of pots was interchanged when refreshing the solution to eliminate the edge effects.

### Gas Exchange and Chlorophyll Fluorescence Measurements

At the end of water stress episode, photosynthetic measurement was conducted on the youngest fully expanded leaves (second from top). In order to ensure that all measurements were conducted under identical conditions, the plants were transferred to a climatic chamber prior to the measurement to acclimate for 1 day in a 12-h photoperiod regime. The climatic chamber was maintained at 30/25°C day/night temperature regime with 800 μmol⋅m^-2^⋅s^-1^ of photosynthetic photon flux density (PPFD) at the top of leaf layer. The CO_2_ concentration in the chamber was about 400 μmol⋅mol^-1^, and the relative humidity was about 70%.

The gas exchange and chlorophyll fluorescence were measured simultaneously using LI-6400XT portable photosynthesis system (Li-Cor Inc., Lincoln, NE, United States) and an integrated fluorescence chamber (6400-40). The initial fluorescence (*F*_o_) and maximum fluorescence (*F*_m_) in dark-adapted state were determined at night. Light-saturated photosynthetic rate was measured from 09:00 to 12:00 with a PPFD of 1500 μmol⋅m^-2^⋅s^-1^, cuvette temperature of 25°C, reference CO_2_ concentration of 400 μmol⋅mol^-1^, and relative humility of 70–80%. Prior to measurement, leaves were placed in the cuvette to acclimate for 10 min. Data were recorded after equilibration to a steady-state. Gas exchange parameters such as net photosynthetic rate (*P*_n_), transpiration rate (*T*_r_), intercellular CO_2_ concentration (*C*_i_), and stomatal conductance (*g*_s_) and fluorescence parameters such as *F*_o_′, *F*_m_′, and *F*_s_ were used for analysis. The following parameters were assessed (Silva et al., 2015): the maximum quantum yield of photosystem II (PSII) photochemistry [*F*_v_/*F*_m_ = (*F*_m_ -*F*_o_)/*F*_m_], the effective quantum yield of PSII photochemistry [Φ_PSII_ = Δ*F*/*F*_m_′ = (*F*_m_′ -*F*_s_)/*F*_m_′], the photochemical quenching coefficient [*qP* = (*F*_m_′ - *F*_s_)/(*F*_m_′ -*F*_o_′)], the non-photochemical quenching coefficient [*NPQ* = (*F*_m_ - *F*_m_′)/*F*_m_′], the apparent electron transport rate at PSII level [*ETR* = Φ_PSII_ × PPFD × 0.5 × α_leaf_], and relative excessive energy at PSII level [*EXC* = (*F*_v_/*F*_m_′) - (Δ*F*/*F*_m_′)/(*F*_v_/*F*_m_)]. To evaluate *ETR*, 0.5 was used as the fraction of excitation energy distributed to PSII, and α_leaf_ was used as the fraction of incoming light absorbed by the leaves. According to Li et al. (2009) and Xiong et al. (2015), the value of α_leaf_ was assumed to be 0.85.

For measurement of *A*-*C*_i_ response curve, the leaves were placed in the cuvette at a PPFD of 1500 μmol⋅m^-2^⋅s^-1^ and CO_2_ concentration of 400 μmol⋅mol^-1^ prior to measurement to equilibrate for 30 min. Cuvette temperature and relative humidity during measurement were maintained as mentioned above. Gas exchange parameters were then recorded in a series of 1000, 800, 600, 400, 300, 200, 150, 100, and 50 μmol⋅mol^-1^ reference CO_2_ concentrations (*C*_a_) when equilibration to a steady-state. Maximum carboxylation rate of Rubisco (*V*_c,max_), maximum electron transport rate (*J*_max_), triose phosphate utilization (*TPU*), and mesophyll conductance (*g*_m_) were calculated as in Sharkey et al. (2007).

After determination of photosynthesis, the measured leaves and similar ones were cut, frozen immediately in liquid nitrogen, and stored at -70°C until use.

### Collection of Xylem Sap

To collect xylem exudation, above-ground parts of plants were cut off from 5 cm above the intersection between the roots and shoots. A known-weight zip-lock bag containing absorbent cotton was covered on the rice stubble for one night (from 18:00 to 8:00) to capture xylem exudation. Then all the bags were collected and sealed and subsequently stored at -20°C. The volume of xylem exudation was calculated as the change of weight of bag divided by the density of xylem exudation, which was assumed to be 1.0 g⋅mL^-1^. The xylem secretion rate was expressed as mL⋅h^-1^.

### Determination of Leaf Relative Water Content

Leaf relative water content (RWC) was estimated as RWC(%) = [(Fw - Dw)/(Sw - Dw)] × 100. Water-saturated weight (Sw) of 0.4 g fresh weight (Fw) leaf samples was obtained by keeping leaf disks in distilled water for 6 h. Then the samples were oven-dried at 70°C to get a constant dry weight (Dw).

### Determination of Chlorophyll Content, Free Amino Acid Content, Proline, and Hydrogen Peroxide Content

Chlorophyll was extracted with about 0.2 g fresh leaf disks by 25 mL mixture of alcohol and acetone (v: v = 1:1) for 24 h in the dark at room temperature. The absorbance of the extract was measured at 663, 645, and 470 nm using a UV-VIS spectrophotometer (UV-2600, Shimadzu, Japan) to estimate chlorophyll *a*, chlorophyll *b* and carotenoids contents according to the method described in Wellburn and Lichtenthaler (1984).

Total free amino acid content was measured chromometrically using ninhydrin method as in Yokoyama and Hiramatsu (2003) and Sun et al. (2006) with some modifications. Amino acids were extracted with acetic acid/sodium acetate buffer (pH5.4), and the content of amino acid was photometric determined at 580 nm. Leucine (Leu) was used as standard. Proline content was determined according to the method in Bates et al. (1973), and L-proline was used as standard.

Hydrogen peroxide (H_2_O_2_) content was measured as in Brennan and Frenkel (1977) and Yang et al. (2007). Samples of frozen leaves (0.3 g) were powdered in liquid N_2_ and homogenized with 5 mL pre-cooled 10 mM 3-amino-1,2,4-triazole. After centrifuging at 8000 rpm for 10 min at 4°C (Centrifuge 5810 R, Eppendorf AG, Germany), 2 mL supernatant and 1 mL 0.1% titanium sulfate in 20% sulfuric acid were added in 10 mL-centrifuge tube. Placed for 10 min, the samples were centrifuged again at 8000 rpm for 10 min. The supernatant was measured colorimetrically at 410 nm. Standard curve was obtained using 30% H_2_O_2_ as standard.

### Soluble Protein, Lipid Peroxidation, and Antioxidase and Glycolate Oxidase Activities

Samples of frozen leaves (0.3 g) were powdered in liquid N_2_ and homogenized with 6 mL sodium phosphate buffer (pH 7.8). The homogenate was centrifuged at 8000 rpm for 10 min at 4°C, then stored at -70°C for the determination of soluble protein and thiobarbituric acid reactive substances (TBARS) contents, and activities of antioxidase and glycolate oxidase (GO). Soluble protein and TBARS were measured according to methods described in Sedmak and Grossberg (1977) and Hodges et al. (1999), respectively. Bovine serum albumin was used as standard in protein assay. TBARS was measured at 600, 532, and 450 nm and calculated using the molar extinction coefficient of 0.155 mM^-1^⋅cm^-1^. The result was expressed with the unit of nmol TBARS⋅g^-1^ Fw.

Superoxide dismutase (SOD) activity was assayed according to the method described in Giannopolitis and Ries (1977). The SOD reaction system contained 25 mmol sodium phosphate buffer (pH7.8), 13 mmol methionine, 2 μmol riboflavin, 10 μmol EDTA-Na_2_, 75 μmol nitro blue tetrazolium (NBT), and modest amount extract. Samples were put under light (300 μmol m^-2^ s^-1^) for 20 min and the absorbance was measured chromometrically at 560 nm.

Ascorbate peroxidase (APX) activity was assayed according to Chen and Asada (1989) with some modifications. The reaction system (3 mL) contained 50 mmol sodium phosphate buffer (pH7.0), 0.3 mmol EDTA-Na_2_, and 0.3 mmol ascorbate acid. The reaction was started by adding 0.06 mmol H_2_O_2_, and the absorbance at 290 nm was monitored for 300 s. APX activity was estimated according to the molar extinct coefficient of ascorbate (2.8 mM^-1^⋅cm^-1^) and expressed as nmol ASA⋅mg^-1^ Pro⋅min^-1^.

Catalase (CAT) activity was determined after the reaction of the extract in the presence of 50 mmol sodium phosphate buffer (pH7.0) and 20 mmol H_2_O_2_ (3 mL). The reaction was carried out at 30°C, and the absorbance at 240 nm was monitored for 300 s (Havir and Mchale, 1987). CAT activity was calculated according to the molar extinct coefficient of H_2_O_2_ (36 mM^-1^⋅cm^-1^) and expressed as nmol H_2_O_2_⋅mg^-1^ Pro⋅min^-1^.

Glycolate oxidase activity was measured by the formation of glyoxylate-phenylhydrazone complex at 324 nm (Baker and Tolbert, 1966). The GO assay mixture (2 mL) contained 50 mmol sodium phosphate buffer (pH7.0, containing 0.1 mmol flavin mononucleotide), 10 mmol phenylhydrazine, 100 mmol L-cysteine, and 0.1 mL extract. The reaction was carried out at 30°C and started with the addition of 100 mmol glycolic acid. The absorbance was monitored for 300 s. The GO activity was calculated using the molar extinction coefficient of the glyoxylate-phenylhydrazone complex (17 mM^-1^ cm^-1^) and expressed as nmol H_2_O_2_⋅mg^-1^ Pro⋅min^-1^.

### Nitrogen Metabolism Enzyme Assay

Samples of 0.3 g frozen leaf were powdered in liquid N_2_ and homogenized with 6 mL 50 mM Tris-HCl buffer (pH = 8.0) containing 2 mM Mg^2+^, 2 mM DTT, and 0.4 M sucrose. The homogenate was centrifuged at 8000 rpm for 10 min at 4°C, and the supernatant was used for determination of the activities of GS, GOGAT, and glutamate dehydrogenase (GDH). The reactions were performed in 3 mL (final volume) of the media indicated below.

Glutamine synthase activity was determined as the method described in Zhang et al. (1997) with some modifications. The reaction mixture contained hydroxylamine hydrochloride buffer (pH7.4). After incubation of the mixture at 37°C for 30 min, the reaction was terminated by adding acidic FeCl_3_ (0.37 M FeCl_3_ and 0.2 M TCA in 0.6 M HCl). Samples were centrifuged at 8000 rpm for 10 min, and the absorbance at 540 nm (A_540_) was measured chromometrically. The blank was absence of hydroxylamine hydrochloride, and the activity of GS was expressed indirectly as A_540_⋅mg^-1^ Pro⋅h^-1^.

Glutamate synthase activity was assayed at 30°C as the method described in Singh and Srivastava (1986). The reaction mixture consisted of 10 μmol α-ketoglutarate, 1 μmol potassium chloride, 37.5 μmol Tris-HCl buffer (pH 7.6), 0.6 μmol NADH, 8 μmol L-glutamine and 0.3 mL enzyme. The absorbance at 340 nm was monitored for 300 s. The activity of GOGAT was estimated using the molar extinction coefficient of NADH (6.22 mM^-1^⋅cm^-1^), and expressed as nmol NADH⋅mg^-1^ Pro⋅min^-1^.

Glutamate dehydrogenase activity was determined as the method described in Loulakakis and Roubelakis-Angelakis (1990). The reaction mixture contained 300 μmol Tris-HCl buffer (pH 8.0), 600 μmol ammonium chloride, 3 μmol calcium chloride, 0.6 μmol NADH, and 0.1 mL enzyme. The reaction was started by adding enzyme extract and carried out at 30°C. The absorbance at 340 nm was monitored for 300 s, and the activity of GDH was expressed as nmol NADH⋅mg^-1^ Pro⋅min^-1^.

### Estimation of Nitrate, Ammonium, and Total Nitrogen

For estimation of nitrate and ammonium, 0.1 g fresh leaf samples were extracted with 25 mL distilled water in boiling water bath for 15 min, and then filtered using ashless filter paper. Nitrate content was determined according to the method described by Cataldo et al. (1975). The reaction mixture consisted of 0.1 mL filtrate and 0.4 mL 5% salicylic acid in concentrated H_2_SO_4_. After 15 min of cooling at room temperature, 9.5 mL 2 M NaOH was added slowly to raise the pH above 12. After cooling the solution at room temperature, absorbance was recorded at 410 nm. Nitrate content was calculated using a calibration curve prepared with KNO_3_ and expressed in mg NO_3_^-^⋅g^-1^ Fw. Ammonium content was measured using the method of indophenol blue colorimetry at 630 nm (Zanini, 2001). Ammonium chloride was used as standard. Fine ground leaf dry samples (0.2 g) were digested with H_2_SO_4_–H_2_O_2_ at 260°C for measurement of total N.

### Statistics

Two-way ANOVAs were conducted to analyze the effects of nitrogen and water. Multiple comparisons were performed using the method of least significant difference (*LSD*) test. Differences were considered statistically significant when *P* < 0.05.

## Results

### Xylem Secretion Rate, Transpiration Rate, and Leaf Relative Water Content

**FIGURE 1:**
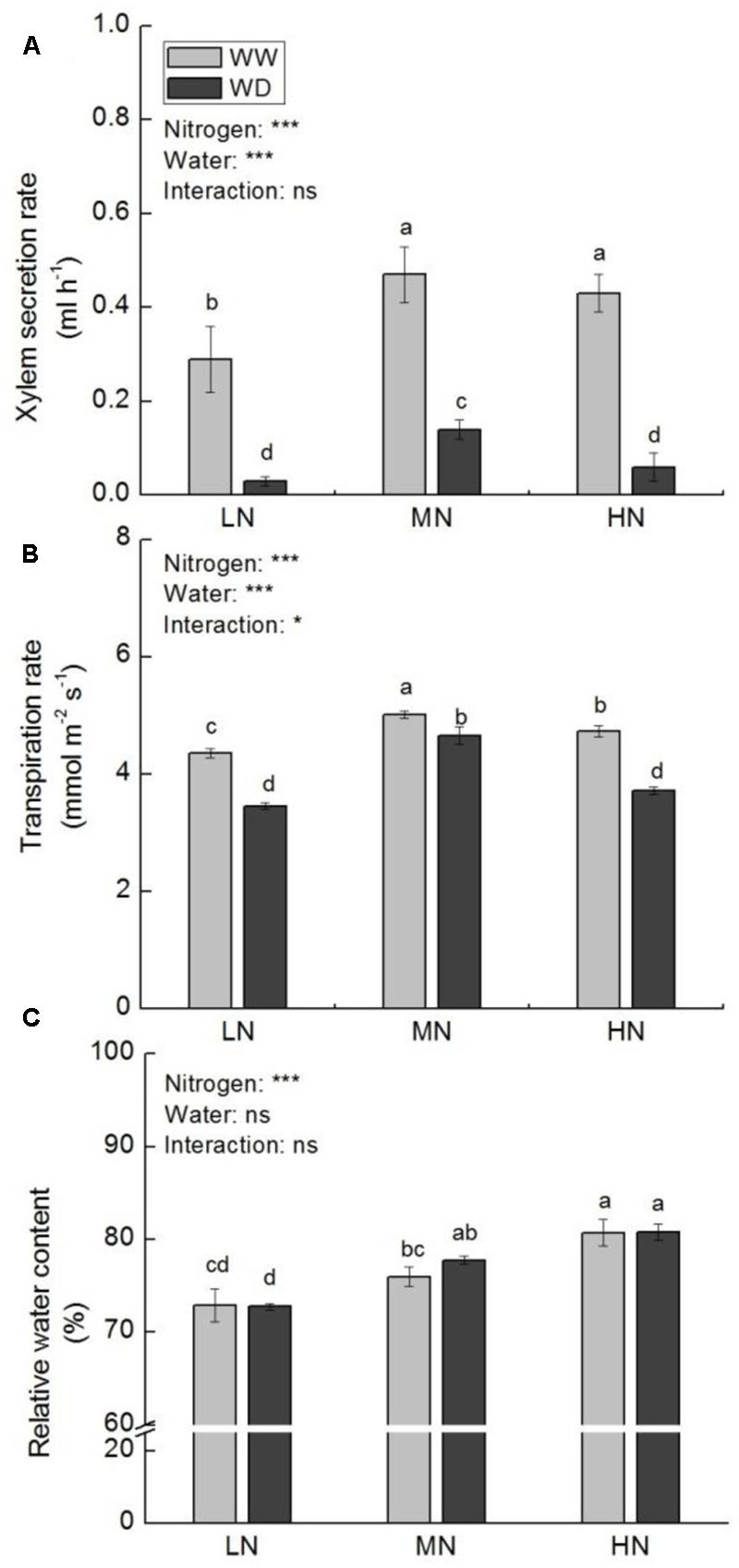
Comparison in xylem secretion rate **(A)**, transpiration rate **(B)**, and leaf relative water content (RWC) **(C)** in rice plants grown under different nitrogen and water conditions. Data refers to mean ± SE (*n* = 4). *P*-values of the two-way ANOVAs of nitrogen, water, and their interaction are indicated: ns, not significant; ^∗^*P* < 0.05; ^∗∗^*P* < 0.01; ^∗∗∗^*P* < 0.001. Bars with the same letter are not significantly different by *LSD* test. WD, water deficit; WW, well-watered.

Relative higher xylem secretion rate, transpiration rate (*T*_r_), and leaf RWC were observed at moderate and high N. Xylem secretion rate and *T*_r_ were significantly suppressed by WD at all N levels, with more reduction at low N (89.9 and 20.6%) and high N (84.9 and 21.4%) versus moderate N (71.0 and 8.6%) (**Figures 1A,B**). However, water condition had no significant effect on RWC at the three N levels (**Figure 1C**).

### CO_2_ Assimilation

**FIGURE 2:**
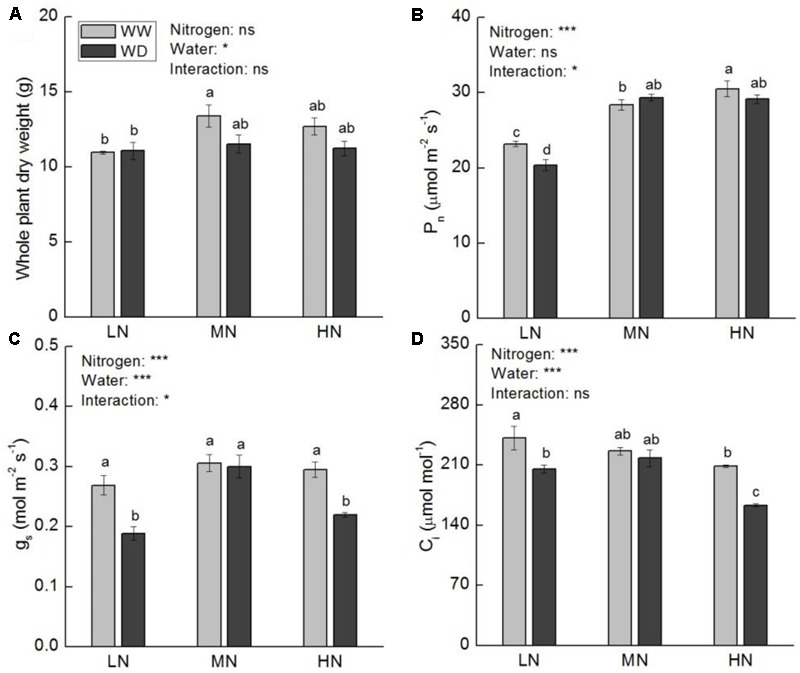
Comparison in whole plant dry weight **(A)**, net photosynthetic rate (*P*_n_, **B**), stomatal conductance (*g*_s_, **C**), and intercellular CO_2_ concentration (*C*_i_, **D**) in rice plants grown udner different nitrogen and water conditions. Data refers to mean ± SE (*n* = 4). *P*-values of the two-way ANOVAs of nitrogen, water, and their interaction are indicated: ns, not significant; ^∗^*P* < 0.05; ^∗∗^*P* < 0.01; ^∗∗∗^*P* < 0.001. Bars with the same letter are not significantly different by *LSD* test. WD, water deficit; WW, well-watered.

**FIGURE 3:**
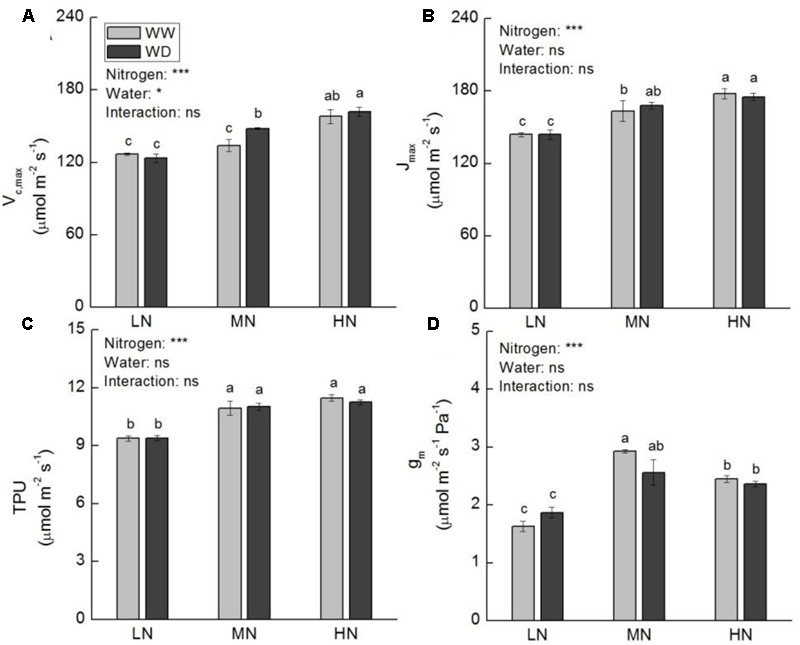
Comparison in maximum carboxylation rate of Rubisco (*V*_c,max_, **A**), maximum electron transport rate (*J*_max_, **B**), triose phosphate utilization (TPU, **C**), and mesophyll conductance (*g*_m_, **D**) of rice plants grown under different nitrogen and water conditions. Data refers to mean ± SE (*n* = 4). *P*-values of the two-way ANOVAs of nitrogen, water, and their interaction are indicated: ns, not significant; ^∗^*P* < 0.05; ^∗∗^*P* < 0.01; ^∗∗∗^*P* < 0.001. Bars with the same letter are not significantly different by *LSD* test. WD, water deficit; WW, well-watered.

Whole plant dry weight (WDW) was not significantly affected by water stress at all three N levels. Nonetheless, a slight reduction in WDW was observed at moderate and high N (**Figure 2A**). Compared to WW, WD significantly decreased *P*_n_, *g*_s_ and *C*_i_ at low N, but these parameters showed no significant differences between WW and WD at moderate N (**Figures 2B–D**). Although WD also significantly reduced *g*_s_ and *C*_i_ at high N, the reduction of *P*_n_ was slight (*P* > 0.05). The *A*-*C*_i_ curve analysis showed that the values of *V*_c,max_, *J*_max_, *TPU*, and *g*_m_ were significant higher at moderate and high N than that at low N (**Figure 3**), irrespective WD or not. However, no significant differences were observed between WW and WD at all N levels except *V*_c,max_ at moderate N, in which water stress increased *V*_c,max_. *V*_c,max_, *J*_max_, and *TPU* were the highest at high N, whereas *g*_m_ was the highest at moderate N.

### Chlorophyll Content and Chlorophyll Fluorescence

**FIGURE 4:**
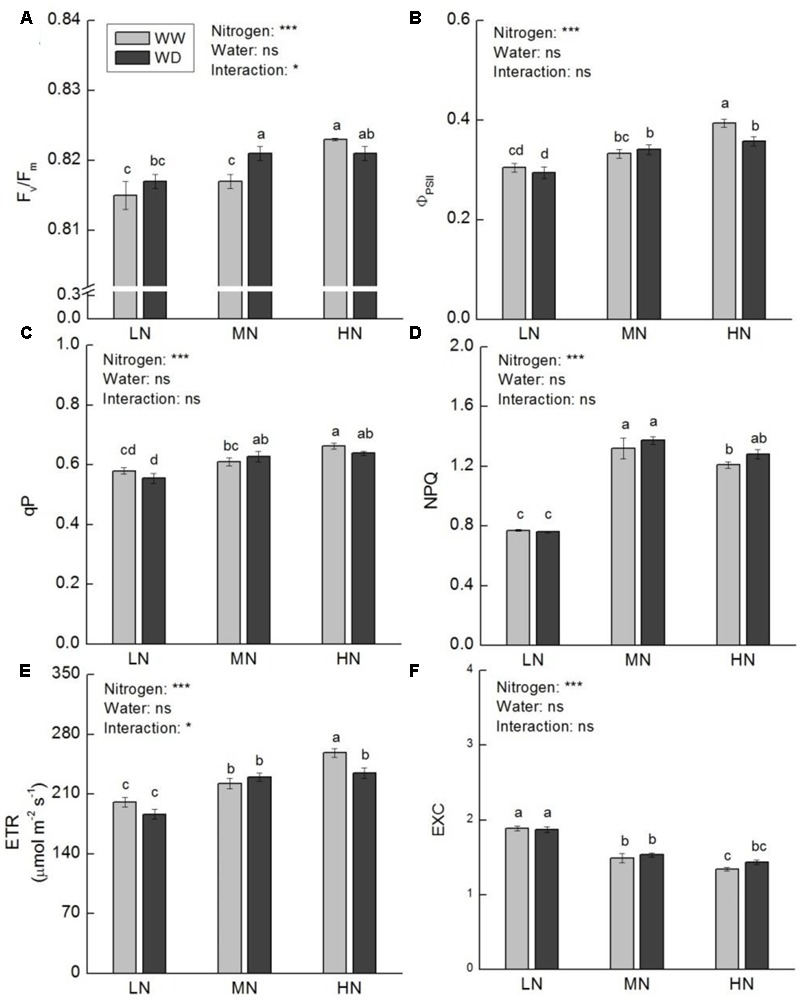
Comparison in chlorophyll fluorescence parameters in rice plants grown udner different nitrogen and water conditions. **(A)** Maximum quantum yield of PSII photochemistry. **(B)** Effective quantum yield of PSII photochemistry. **(C)** Photochemical quenching coefficient. **(D)** Non-photochemical quenching coefficient. **(E)** Electron transport rate at PSII level. **(F)** Relative excessive energy at PSII level. Data refers to mean ± SE (*n* = 4). *P*-values of the two-way ANOVAs of nitrogen, water, and their interaction are indicated: ns, not significant; ^∗^*P* < 0.05; ^∗∗^*P* < 0.01; ^∗∗∗^*P* < 0.001. Bars with the same letter are not significantly different by *LSD* test. WD, water deficit; WW, well-watered.

Nitrogen had considerable effect on chlorophyll content, whereas WD showed no significant effect on leaf chlorophyll content at low and high N (**Table 1**). However, WD significantly decreased leaf total chlorophyll, chlorophyll *a* and carotenoid contents at moderate N. To evaluate the influence of chlorophyll on photosynthesis, chlorophyll fluorescence was further measured. The results in **Figure 4** displayed that *F*_v_/*F*_m_, Φ_PSII_, *qP, NPQ*, and *ETR* were higher at moderate and high N. It is noteworthy that *NPQ* at moderate and high N was ∼0.5-fold higher than that at low N irrespective water stress or not. In contrast, *EXC* was lower at moderate and high N. Water scarcity merely caused a significant increase in *F*_v_/*F*_m_ at moderate N, and a significant reduction in Φ_PSII_ and *ETR* at high N.

**Table 1:** Comparison in chlorophyll contents of rice plants grown under different nitrogen and water conditions.

Treatment		Chlorophyll *a* content/mg g^-1^ Fw	Chlorophyll *b* content/mg g^-1^ Fw	Carotenoid content/mg g^-1^ Fw	Total chlorophyll content/mg g^-1^ Fw
Low N	WW	2.83 ± 0.04 c	0.82 ± 0.01 c	0.59 ± 0.01 b	4.23 ± 0.05 d
	WD	2.90 ± 0.02 c	0.85 ± 0.02 c	0.61 ± 0.01 b	4.36 ± 0.04 d
Moderate N	WW	3.43 ± 0.12 b	1.23 ± 0.02 b	0.69 ± 0.03 a	5.35 ± 0.14 b
	WD	2.94 ± 0.09 c	1.44 ± 0.07 a	0.54 ± 0.03 b	4.92 ± 0.11 c
High N	WW	3.69 ± 0.09 a	1.45 ± 0.07 a	0.72 ± 0.02 a	5.86 ± 0.08 a
	WD	3.64 ± 0.06 ab	1.48 ± 0.03 a	0.72 ± 0.02 a	5.84 ± 0.04 a
	Nitrogen	52.75^∗∗∗^	115.27^∗∗∗^	17.13^∗∗∗^	166.73^∗∗∗^
*F*-value	Water	5.95^∗^	6.55^∗^	5.87^∗^	2.42 ns
	Nitrogen × water	7.17^∗∗^	2.73 ns	8.52^∗∗^	5.72^∗^

*Data refers to mean ± SE (*n* = 4). *F*-values of the two-way ANOVAs of nitrogen, water, and their interaction are indicated: ns, not significant; ^∗^*P* < 0.05; ^∗∗^*P* < 0.01; ^∗∗∗^*P* < 0.001. Means in the same column with the same letter are not significantly different by *LSD* test. WD, water deficit; WW, well-watered.*

### Nitrogen Compounds and Activities of Nitrogen Metabolism Enzymes

**FIGURE 5:**
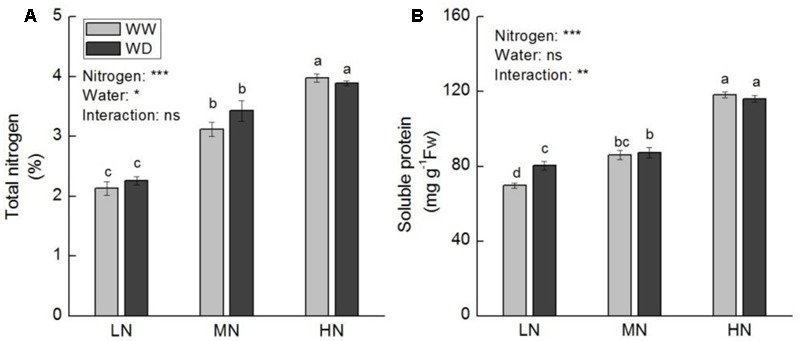
Comparison in the contents of total leaf nitrogen **(A)** and soluble protein **(B)** in rice plants grown udner different nitrogen and water conditions. Data refers to mean ± SE (*n* = 4). *P*-values of the two-way ANOVAs of nitrogen, water, and their interaction are indicated: ns, not significant; ^∗^*P* < 0.05; ^∗∗^*P* < 0.01; ^∗∗∗^*P* < 0.001. Bars with the same letter are not significantly different by *LSD* test. WD, water deficit; WW, well-watered.

**FIGURE 6:**
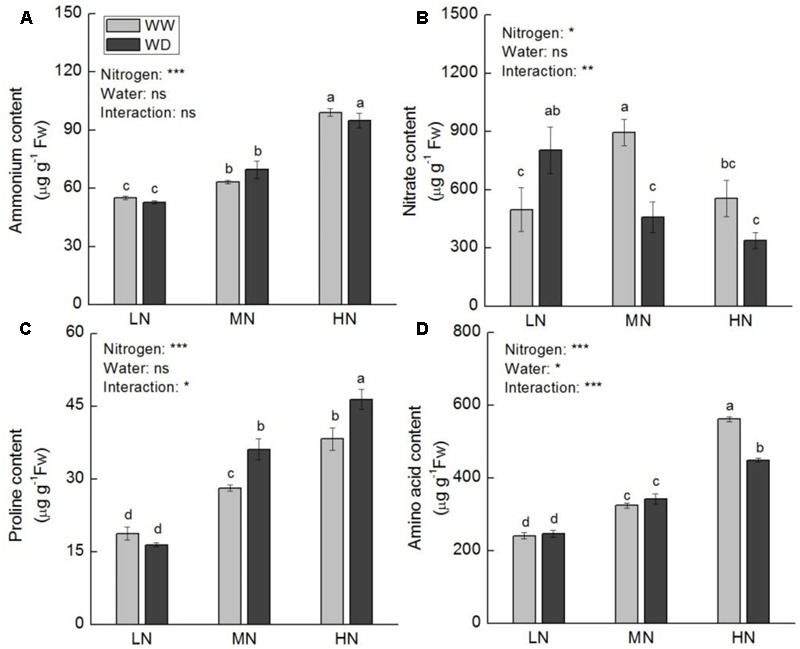
Comparison in the contents of ammonium **(A)**, nitrate **(B)**, proline **(C)**, and free amino acid **(D)** in rice plants grown udner different nitrogen and water conditions. Data refers to mean ± SE (*n* = 4). *P*-values of the two-way ANOVAs of nitrogen, water, and their interaction are indicated: ns, not significant; ^∗^*P* < 0.05; ^∗∗^*P* < 0.01; ^∗∗∗^*P* < 0.001. Bars with the same letter are not significantly different by *LSD* test. WD, water deficit; WW, well-watered.

Contents of total leaf N and major nitrogenous compounds (soluble protein, ammonium, proline, and free amino acids) increased with increasing N level. Total N, soluble protein and ammonium showed no significant difference between WD and WW at the three N levels (except soluble protein at low N) (**Figures 5, 6A**). However, WD increased nitrate content significantly at low N, but decreased nitrate content significantly at moderate and high N (**Figure 6B**). Contrary to nitrate, WD increased leaf proline content considerably at moderate N and high N, while slightly decreased proline content at low N, compared to WW (**Figure 6C**). Leaf free amino acid content was not significantly affected by WD at low and moderate N, while it was substantially reduced by WD at high N (**Figure 6D**).

**FIGURE 7:**
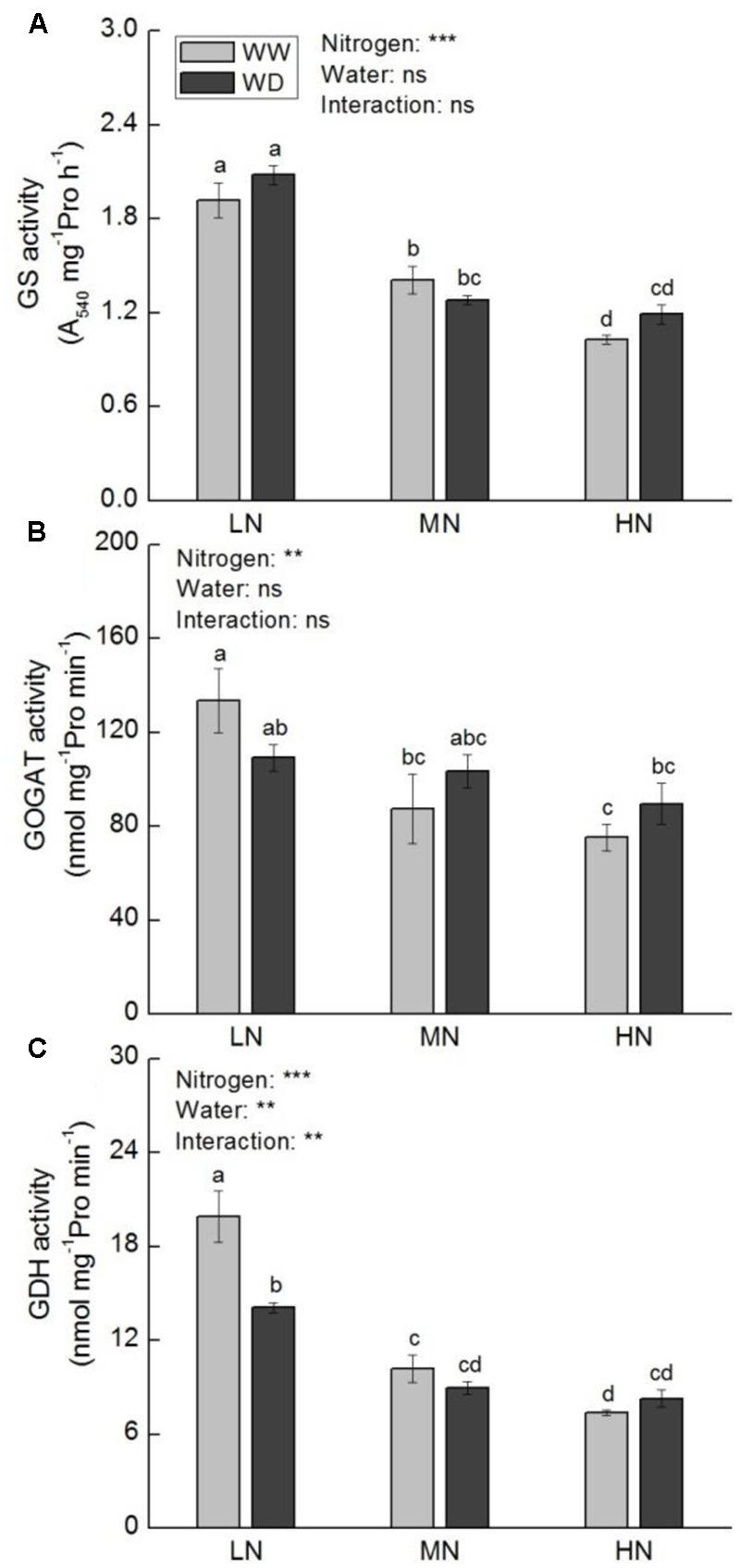
Comparison in activities of glutamine synthase (GS, **A**), NADH-glutamate synthase (NADH-GOGAT, **B**), and NADH-glutamate dehydrogenase (NADH-GDH, **C**) in rice plants grown under different nitrogen and water conditions. Data refers to mean ± SE (*n* = 4). *P*-values of the two-way ANOVAs of nitrogen, water, and their interaction are indicated: ns, not significant; ^∗^*P* < 0.05; ^∗∗^*P* < 0.01; ^∗∗∗^*P* < 0.001. Bars with the same letter are not significantly different by *LSD* test. WD, water deficit; WW, well-watered.

Under WW condition, GS, GOGAT, and GDH activities were decreased with increasing N level (**Figure 7**). Water scarcity reduced the activities of GOGAT (*P* > 0.05) and GDH (*P* < 0.05) at low N. whereas their activities were slightly reduced or even increased by WD at moderate and high N. The activity of GS was not significantly affected by WD at all N levels.

### Oxidative Damage and Activities of Ascorbate Peroxidase, Catalase, Superoxide Dismutase, and Glycolate Oxidase

**FIGURE 8:**
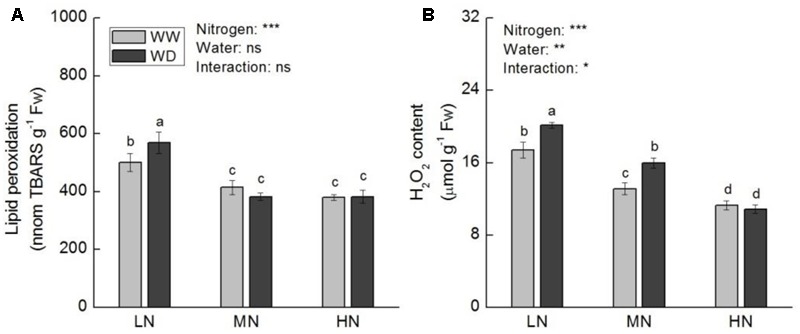
Comparison in leaf lipid peroxidation **(A)** and hydrogen peroxide **(B)** in rice plants grown udner different nitrogen and water conditions. Data refers to mean ± SE (*n* = 4). *P*-values of the two-way ANOVAs of nitrogen, water, and their interaction are indicated: ns, not significant; ^∗^*P* < 0.05; ^∗∗^*P* < 0.01; ^∗∗∗^*P* < 0.001. Bars with the same letter are not significantly different by *LSD* test. WD, water deficit; WW, well-watered.

Lipid peroxidation and H_2_O_2_ content were decreased as the increase of N level, regardless WD or not (**Figure 8**). At low N, WD induced significant increases in lipid peroxidation and H_2_O_2_ content, while no significant differences were observed between WW and WD at moderate N (excepted H_2_O_2_ content) and high N.

**FIGURE 9:**
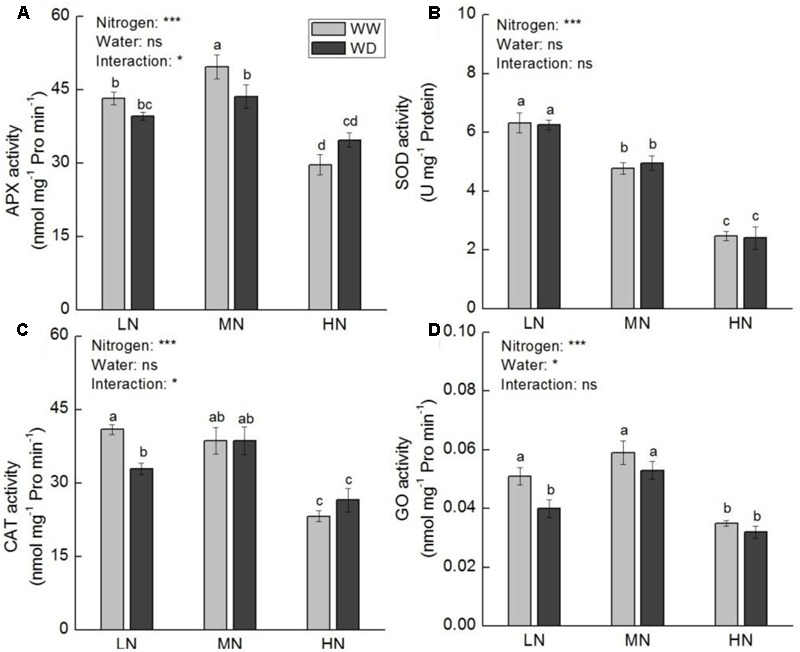
Comparison in activities of ascorbate peroxidase (APX, **A**), superoxide dismutase (SOD, **B**), catalase (CAT, **C**), and glycolate oxidase (GO, **D**) in rice plants grown udner different nitrogen and water conditions. Data refers to mean ± SE (*n* = 4). *P*-values of the two-way ANOVAs of nitrogen, water, and their interaction are indicated: ns, not significant; ^∗^*P* < 0.05; ^∗∗^*P* < 0.01; and ^∗∗∗^*P* < 0.001. Bars with the same letter are not significantly different by *LSD* test. WD, water deficit; WW, well-watered.

Activities of APX, SOD, CAT, and GO at low and moderate N were higher relative to that at high N, irrespective WD or not (**Figure 9**). WD reduced the activities of APX (*P* > 0.05), CAT (*P* < 0.05) and GO (*P* < 0.05) at low N, but increased CAT and APX activities at high N (*P* > 0.05). SOD activity decreased with the increasing N level, with no significant difference between WW and WD.

## Discussion

### Effects of Water Stress on Photosynthesis at Different N Levels

It has been widely verified that plant biomass or yield is not always in line with photosynthetic rate of single leaf. Our results showed that plant growth was slightly inhibited by water stress at moderate and high N, whereas their photosynthesis was not affected. The different sensitivity of plant growth and photosynthesis to water stress could contribute to the different performances of biomass and photosynthetic rate in response to water stress. Besides, the trade-off between growth and stress defense could also be responsible for the resistance of photosynthesis to water stress (Karasov et al., 2017). The significant effect of nitrogen and water stress interaction on photosynthetic rate implies that N is essential for regulating the adaptation of photosynthesis to water stress.

Water stress tolerance of plant is significantly governed by its water retention capacity (Gill et al., 2015). Transpiration is the major pathway of water loss from leaves. Generally, plants reduce water loss by partial closing of stomata under water stress conditions. In accordance with xylem secretion rate, transpiration rate was significantly reduced under water stress in this study. As a result, leaf RWC was maintained even under water stress. It has been suggested that higher water acquisition capacity under water stress may contribute to higher photosynthetic rate (Guo et al., 2007; Gao et al., 2010). Although water scarcity reduced active water absorption of roots in this study, it remained higher water acquisition capacity at higher N (**Figure 1**). This may result from N-mediated higher root activity. It has been reported that higher N application increased rice root activity (Xu et al., 2015). In another study, we also observed that rice root activity was higher at high N even in the situation of water stress (data not published).

Stomata control water and gas exchange between leaves and ambient air. Trade-off between water loss and gas exchange is crucial for plants to maintain photosynthetic capacity under WD condition (Manzoni et al., 2013). Although partial closure of stomata is paramount for prohibiting water loss to maintain water balance in plant, stomatal closure increases the resistance of CO_2_ diffusion from ambient air to intercellular airspace (*g*_s_), as well as from intercellular airspace to carboxylation site (*g*_m_). To date, the relative importance of *g*_s_ and *g*_m_ in photosynthesis remains controversial (Niinemets et al., 2009; Xiong et al., 2015; Barbour and Kaiser, 2016). In the current study, *g*_s_ could be more important than *g*_m_ for regulating photosynthesis under water stress. Water stress and its interaction with nitrogen showed significant effects on *g*_s_, and a stomatal limitation to photosynthesis occurred at low N, suggesting that N is a pivotal factor in regulating stomata movement under water scarcity. Higher N could increase the sensitivity of stomata to water stress to prohibit (or alleviate) stomatal limitation of photosynthesis (Otoo et al., 1989). Hacke et al. (2010) proposed that aquaporin may take part in the regulation of N on *g*_s_. However, more studies are needed to identify the role that N plays in the regulation of stomatal movement. It is speculated that *g*_m_ is not the main factor affecting photosynthetic rate under water stress in this study, as it was affected by nitrogen rather than water deficiency. *g*_m_ is determined by both complex leaf anatomical structure and biochemical factors (Giuliani et al., 2013). It is postulated that the unchanged *g*_m_ under water stress could be due to the fact that short-term WD treatment is not long enough to change the leaf anatomical structure. Besides, the quick restoration of *g*_m_ during the acclimation of water stress may also result in the undifferentiated *g*_m_ between water treatments (Galle et al., 2010).

*V*_c,max_, *J*_max_, and *TPU* reflect the three biochemical factors limiting photosynthesis under light-saturation condition: (i) the amount and activity of Rubisco; (ii) the regeneration rate of RuBP; and (iii) the utilization of triose phosphate and regeneration of Pi for photophosphorylation, respectively (Farquhar et al., 1980; von Caemmerer and Farquhar, 1981). Rubisco represents the majority of N invested in photosynthesis. It can excess a quarter of leaf N and account for as much as half of soluble protein (Parry et al., 2013). It is the rate-limiting enzyme of CO_2_ fixation in C_3_ plants. It has been reported that *V*_c,max_ was affected by water stress only when the stress becomes severe (Bota et al., 2004; Rivero et al., 2009; Galle et al., 2010). Our results suggest that Rubisco could be one of the factors affecting photosynthetic rate even under moderate water stress. Furthermore, the effect of water stress on *V*_c,max_ is N level related; higher N is beneficial to increase or maintenance of Rubisco activity under moderate water scarcity condition.

It should be noted that WD substantially reduced *g*_s_ and *C*_i_ at high N as well. Concurrently, the decrease of Φ_PSII_ and ETR were also observed at high N. Due to *P*_n_ was not affected in WD, the reduction of Φ_PSII_ and *ETR* could be an adaptive strategy of photosynthesis rather than an indicator of damage in response to water stress.

### Effects of Water Stress on N Assimilation at Different N Levels

Nitrogen assimilation is tightly linked with carbon metabolism in the fundamental biochemical pathways in plants. N assimilation, especially the reduction of nitrate, is a highly energy dependent reaction (Bloom et al., 2010). Incorporation of inorganic N into amide and amino acid consumes energy and reducing equivalents produced in photosynthesis, mitochondrial oxidative metabolism, or other cytosolic reactions, depending on the site of reaction (Sunil et al., 2013). Reduction of nitrate in leaves could use the excessive reducing power derived from photosynthesis, and it is more efficient than the reduction which takes place in roots in water stress situation (Gonzalez-Dugo et al., 2010). Thus, N assimilation acts as an important alternative sink of electron and excessive excited energy to minimize photoinhibition and photodamage of photosynthesis, and to stimulate CO_2_ assimilation under conditions of stomatal limitation imposed by osmotic stress (Yi et al., 2014).

The effects of water stress on leaf N status are erratic, with increase, decrease, or constant in different species (Jangam and Raghuram, 2015). In this study, total leaf N content was not affected by water stress at all the three N levels; however, water stress induced alterations of some nitrogenous compounds (e.g., nitrate, protein, proline, and amino acid) in rice leaf were N level dependent. Nitrate is an important N pool in plant (Han et al., 2016). When N supply is sufficient, a considerable amount of nitrate is stored in vacuoles, which can be reutilized when N supply is limited (e.g., water stress induced decrease of N uptake). The result in this study suggests that sufficient N supply facilitates assimilation of stored nitrate under WD condition, which could partly contribute to mitigating photoinhibition of photosynthesis caused by water stress. Water stress can lead to proteolytic breakdown of protein and consequently result in accumulation of amino acids (Akinci and Sösel, 2012). However, water stress did not lead to a decline in soluble protein in our study. The variation of amino acids resulted from water stress at different N levels is more likely to reflect the changes in N uptake and assimilation, and turnover of amino acid, e.g., proline synthesis (Walch-Liu et al., 2005). It is generally accepted that proline is a preferred soluble organic osmolyte in many plants, as well as an important contributor to stabilize membrane structure by buffering cellular redox potential (Szabados and Savouré, 2010; Singh et al., 2016). Nevertheless, the significance of proline accumulation in osmotic adjustment is not always apparent (Szabados and Savouré, 2010). In some studies, accumulation of proline seems to be a symptom of injury rather than an indicator of stress tolerance (Hoai et al., 2003). While in this study, water stress induced increase in proline content at moderate and high N serves more likely as an osmotica to protect rice photosynthesis against water stress. The adverse changes of nitrate and proline induced by water stress further suggest that sufficient N supply provides substantial foundation for improving the resistance of photosynthesis to water stress in rice, which may rely greatly on N assimilation.

Ammonium acts at the center of N flow in plant leaf (Thomsen et al., 2014). Given a large amount of ammonium is produced as a result of protein hydrolysis and photorespiration, it is essential that toxic ammonium be immediately reassimilated into organic molecules for nitrogen cycling (Masclaux-Daubresse et al., 2006). GS, GOGAT, and GDH are important enzymes involving in N recycling in plant. It has been widely reported that photosynthesis and activities of key enzymes related to nitrogen assimilation were consistently down-regulated under water stress condition (Garg et al., 2001; Xu and Zhou, 2006; Pinheiro and Chaves, 2011). However, our results displayed that the response of activities of N assimilation enzymes to water stress was N level dependent. These enzymes were more sensitive to water stress at low N, and water stress had a greater effect on GDH than on GS and GOGAT. Since GDH and GS–GOGAT pathway play distinct roles in N cycle (Masclaux-Daubresse et al., 2006), it is implied that other N metabolic processes besides primary N assimilation are regulated by water stress. In addition to ammonium assimilation, Boussama et al. (1999) proposed the function of GDH in the synthesis of proline by providing Glu under stress condition. Water stress induced increase in soluble protein and decrease in proline at low N (**Figures 5B, 6C**) could be partially resulted from the decline in GDH activity. It is suggested that GDH is essential for the acclimation of rice photosynthesis to water stress. GO is a key enzyme in photorespiration, which catalyzes the oxidation of glycolate to glyoxylate. The latter is in turn converted to glycine by transamination. Glycine is further converted to serine in mitochondria by transamination to release ammonia. It is well known that in non-leguminous C_3_ plants, the flux of ammonia released during photorespiration could be more than 10-fold the rate of primary assimilation (Masclaux-Daubresse et al., 2006). The photorespiratory N cycle contributes to the metabolism of certain amino acids, e.g., Gln, Glu, Ser, and Gly (Igarashi et al., 2006). Decrease in photorespiration will lead to decline in photosynthesis and nitrate assimilation (Bloom et al., 2010; Silva et al., 2015). The results of GO activity together with N assimilation enzymes and nitrogenous compounds reveal that nitrate and ammonium assimilation play important roles in adaption of rice photosynthesis to water stress, protecting photosynthesis from inhibition by water scarcity.

### Effects of Water Stress on Antioxidant Capacity at Different N Levels

Water stress induced disequilibrium between light energy absorption and utilization will cause the accumulation of ROS, and consequently increase the photooxidative damage to photosynthesis, performed as decline in *P*_n_ and enhancement in peroxidation of cell membrane (Hassan et al., 2012; Gill et al., 2015). Therefore, maintenance of appropriate level of ROS in cell and prevention of irreversible damage to photosynthetic machinery is essential for regular photosynthesis (Chaves and Oliveira, 2004; Sunil et al., 2013). There are multiple effective mechanisms to avoid excessive accumulation of ROS in plants, including avoidance of excessive light energy absorption, enhancement of energy dissipation, and improvement of antioxidant systems (Silva et al., 2015; Chang et al., 2016). Studies with pigment mutants revealed that photosynthetic pigment reduction does not necessarily lead to decline in photosynthetic rate. Reduced pigment content could enable plants to avoid excessive light energy absorption and improve quantum efficiency of PSII and electron transport rate, leading to higher photosynthetic rate (Wu et al., 2014; Gu et al., 2017). Reduced *F*_v_/*F*_m_ reflects the photoinhibition of PSII (Misra et al., 2012). In this study, the reduced chlorophyll content and increased *F*_v_/*F*_m_ at moderate N in response to water stress indicate that alteration in chlorophyll content is an efficient strategy to prevent photosynthesis from photoinhibition. *NPQ* is a common mechanism that plant dissipates excessive excited energy under high light, high N, and water stress. The increased *NPQ* with N level was in parallel with decrease in TBARS and H_2_O_2_ contents, suggesting that *NPQ* is important for preventing photosynthesis from photodamage. Water stress can modulate activities of antioxidant enzymes in plants (Gill et al., 2015). In addition, plant antioxidant capacity is depended greatly upon N availability. Higher N improves stress tolerance of plants via enhancement of the antioxidant ability and inhibition of lipid peroxidation (Fu and Huang, 2003). On the contrary, low N attenuates ROS scavenging system and increases oxidative stress in leaves (Ramalho et al., 1998). Our results are consistent with those findings that low N led to significant higher lipid peroxidation and H_2_O_2_ content compared to moderate and high N. Besides, water scarcity aggravated oxidative injure to membrane at low N. Xu et al. (2014) proposed that proline could protect antioxidative enzymes. Similarly, in this study, the responses of CAT and APX activities to water stress were consistent with proline content (except moderate N). The results indicate that higher level of N metabolism may have contributed to water stress tolerance of photosynthesis in rice by preventing cell membrane damage, and this could as a result of effective energy dissipation and ROS scavenging system.

Photorespiration is a significant and important component of physiological process overlapping other ways of primary metabolism (Peterhansel and Maurino, 2011). The reactions in photorespiration, e.g., the conversion of hydroxypyruvate to glycerate and the subsequent to PGA, and the re-fixation of NH_4_^+^ deriving from glycine decarboxylation, require energy (ATP) and reducing equivalents [NAD(P)H] (Peterhansel and Maurino, 2011). Accordingly, photorespiration is recognized as a vital mechanism for mitigation of ROS generation and photoinhibition that can occur under water stress condition (Guan and Gu, 2009; Peterhansel et al., 2010; Zhang et al., 2016). CAT has positive feedback on GO activity, for CAT scavenges H_2_O_2_ derived from GO catalyzed conversion of glycolate to glyoxylate (Zelitch, 1990). The significant reduction in GO activity at low N could be partially by virtue of reduced CAT activity.

## Conclusion

Our findings reveal that higher N increases adaptability of rice photosynthesis to water stress via the pathways including: (i) mitigation of stomatal limitation to photosynthesis and maintenance of higher Rubisco activity; (ii) maintained or increased antioxidant enzymes activities and higher excessive energy dissipation capacity; and (iii) increased nitrate and ammonium assimilation and synthesis of proline. Nitrogen metabolism plays a central role in these pathways. The results suggest that the resistance of rice photosynthesis to WD could be improved by manipulation of key enzymes involving in N metabolism.

## Author Contributions

CZ collected the samples, analyzed the samples, and drafted the manuscript. XC made a contribution to design of the work and to draft the manuscript. JjH collected the samples and analyzed the samples. LZ and JZ analyzed the data and revised the manuscript. JlH and QJ conceived and designed this work. All authors read and approved the manuscript.

## Conflict of Interest Statement

The authors declare that the research was conducted in the absence of any commercial or financial relationships that could be construed as a potential conflict of interest. The reviewer JJR and handling Editor declared their shared affiliation, and the handling Editor states that the process met the standards of a fair and objective review.
